# Strategies For Enhancing Equity, Diversity, and Inclusion in Medical School Admissions–A Canadian Medical School's Journey

**DOI:** 10.3389/fpubh.2022.879173

**Published:** 2022-06-24

**Authors:** Tisha R. Joy

**Affiliations:** Department of Medicine, Schulich School of Medicine and Dentistry, Western University, London, ON, Canada

**Keywords:** diversity, equity, inclusion, medical school admissions, Canada, physician workforce

## Abstract

**Background:**

Medical schools aim to select and train future physicians representative of and able to serve their diverse population needs. Enhancing equity, diversity, and inclusion (EDI) in admissions processes includes identifying and mitigating barriers for those underrepresented in medicine (URM).

**Summary of Innovations:**

In 2017, Schulich School of Medicine and Dentistry (Western University, Ontario, Canada) critically reviewed its general Admissions pathways for the Doctor of Medicine (MD) program. Till that time, interview invitations were primarily based on academic metrics rather than a holistic review as for its Indigenous MD Admissions pathway. To help diversify the Canadian physician workforce, Schulich Medicine utilized a multipronged approach with five key changes implemented over 2 years into the general MD Admissions pathways: 1. A voluntary applicant diversity survey (race, socioeconomic status, and community size) to examine potential barriers within the Admissions process; 2. Diversification of the admissions committee and evaluator pool with the inclusion of an Equity Representative on the admissions committee; 3. A biosketch for applicants' life experiences; 4. Implicit bias awareness training for Committee members, file reviewers and interviewers; and 5. A specific pathway for applicants with financial, sociocultural, and medical barriers (termed ACCESS pathway). Diversity data before (Class of 2022) vs. after (Class of 2024) these initiatives and of the applicant pool vs. admitted class were examined.

**Conclusion:**

For the Class of 2024, the percentage of admitted racialized students (55.2%), those with socioeconomic challenges (32.3%), and those from remote/rural/small town communities (18.6%) reflected applicant pool demographics (52.8, 29.9, and 17.2%, respectively). Additionally, 5.3% (vs. 5.6% applicant pool) of admitted students had applied through ACCESS. These data suggest that barriers within the admissions process for these URM populations were potentially mitigated by these initiatives. The initiatives broadly improved representation of racialized students, LGBTQ2S+, and those with disability with statistically significant increases in representation of those with socioeconomic challenges (32.3 vs. 19.3%, *p* = 0.04), and those with language diversity (42.1 vs. 35.0%, *p* = 0.04). Thus, these changes within the general MD admissions pathways will help diversify the future Canadian physician workforce and inform future initiatives to address health equity and social accountability within Canada.

## Introduction

Health disparities based on race, income, immigrant status, LGBTQ2S+ identity, and disability exist in both Canada and the United States (1–4). In order to promote social accountability and improve health equity, enhancing physician workforce diversity has been an ongoing priority in these countries (5–7) since physician workforce diversity has been demonstrated to strengthen the patient-physician relationship, increase patient satisfaction and trust, improve adherence to recommendations and patient-related health care outcomes, as well as broaden access to health care resources particularly for underserved and vulnerable populations (8–14).

## Background and Rationale

Physicians who are underrepresented in medicine (URM) may include those who self-identify as Indigenous, racialized, female, LGBTQ2S+, as well as those from socioeconomically-challenged backgrounds [low socioeconomic status (SES)], persons with disabilities [PWD], and/or those from rural upbringings. Physicians who belong simultaneously to more than one URM population possess intersectionality, which may be an important consideration in empathy and care for underserved populations. Several studies have identified that physicians who belong to a URM population are more likely to care for patients from underserved populations (15–18). Thus, recruiting diverse medical students may be helpful to the goal of improving health equity. Moreover, Saha et al. (18) demonstrated that belonging to a racially and ethnically diverse medical class resulted in non-racialized medical students feeling better prepared to care for racialized patient populations. Thus, enhancing medical student diversity not only improves physician workforce diversity directly but also enhances the cultural comfort of the entire medical school class to ultimately better serve the needs of their diverse patient populations.

The Canadian population is 4.9% Indigenous, 50.9% female, 4% LGBTQ2S+, and 22.3% racialized (19, 20). Approximately 11% of the Canadian population have low-income backgrounds and 31% reside in small towns and/or rural areas (21, 22). Twenty-four percent are first generation Canadians (i.e., having immigrated to Canada) (19). Although the official languages in Canada are English and French, 21% of the Canadian population speak a first language other than these (19). Of Canadians aged 15 years or older, ~22% have one or more disabilities (23). Thus, given the diversity amongst the Canadian population, there is a growing need for medical schools to select and train future physicians representative of and able to serve the needs of these various populations. Within Canada, there is underrepresentation of physicians who are Indigenous, racialized (especially Black, Filipino), of rural origin, with disabilities, and with socioeconomically challenged backgrounds (24–26). Physician data on diversity parameters such as sexual identity, immigration status, and language diversity are lacking (24–27).

## Context

Schulich School of Medicine and Dentistry (Schulich Medicine) at Western University located in London, Ontario is one of the 17 medical schools within Canada that can play a vital role in enhancing Canadian physician workforce diversity. In the fall of 2017, Schulich Medicine undertook a critical review of its medical school admissions processes to determine where improvements could be made to fulfill this need. Since Schulich Medicine already had a longstanding pathway specifically for self-identifying Indigenous applicants grounded in a holistic approach, the admissions committee focused its initial efforts on identifying barriers and developing initiatives to ensure equity for URM applicants within its non-Indigenous general admissions pathways to the Doctor of Medicine (MD) program.

The Schulich Medicine admissions committee took a broad approach to enhancing the diversification of the Canadian physician workforce within its non-Indigenous pathways and defined URM physicians as those who self-identify as racialized, female, LGBTQ2S+, PWD, as well as those from socioeconomically-challenged backgrounds, and/or those from rural upbringings. In addition, life experience, educational diversity, and language diversity were believed to contribute to the overall learning within the medical school cohort and the care that could potentially be provided for Canada's multicultural and multigenerational population. Thus, Schulich Medicine decided to additionally focus and track metrics related to the percentage (%) of mature students (defined as those aged 25 years or older), language diversity (those who spoke a first language other than English or French), and educational diversity (graduate students, those who are first generation to attend University, and those who are first generation to enter medical school) within its incoming medical class.

To encourage other schools to consider adopting a multipronged approach to fostering equity, diversity, and inclusion (EDI), this paper will discuss the initiatives implemented at Schulich Medicine within 2 years of its critical review and explore the impact of this multipronged approach on the diversity of the medical school class that entered in fall 2020 [Class of 2024 (i.e., post-initiatives)]. First, we will compare the diversity parameters of the applicant pool of 2019–2020 to those of the incoming Class of 2024 to determine the impact of the initiatives on barriers within the admission process itself. And second, we will examine the diversity metrics of the MD Class of 2024 (post-initiatives) to those of the MD Class of 2022 [those who entered in fall 2018 (pre-initiatives)].

## Essential Elements to Enhancing EDI in Medical School Admissions

### Critical Review of Admissions Processes to Identify Gaps and Barriers

In fall 2017, the admissions committee examined historical changes to admissions requirements/processes as well as its current application and evaluation processes informed by the Schulich Medicine diversity statement (28). It then conducted an environmental scan of admissions requirements and processes across various Canadian and US medical schools, including the components of a holistic review as outlined by the Association of American Medical Colleges (AAMC) (29) and literature regarding strategies for enhancing diversity, mitigating bias, and assessing non-academic attributes (30–32).

Based on this information, the admissions committee identified several initiatives pre-2017 that were likely already useful to fostering EDI within the medical school admissions process, such as the use of only two undergraduate years in full-time studies for grade-point average (GPA) calculation, the lack of course pre-requisites (e.g., Biology, Physics) given its use of the Medical College Admission Test (MCAT), the lack of a preferred undergraduate degree (e.g., Science) or preferred university for undergraduate degree completion. These steps encouraged students to pursue undergraduate studies of their own interest, promoted educational and cognitive diversity within the admitted medical school class while aiming for a baseline assessment of pre-medical knowledge, and recognized that full-time studies throughout an undergraduate degree may not be feasible for all students (e.g., those from socioeconomically challenged backgrounds, PWD]). Furthermore, Schulich Medicine already had two medical school Admissions pathways prioritizing social accountability, equity, and diversity: 1. the Indigenous admissions pathway for applicants who self-identify as Indigenous, and 2. the Southwestern Ontario (SWO) pathway for applicants who had completed all four high school years within the predominantly rural catchment region surrounding Schulich Medicine. Applicants who did not qualify for these two Admissions pathways were considered within the general Admissions pathway, which typically comprised the vast majority (~85–90%) of applicants.

Applicants to the six medical schools within Ontario (Canada), including Schulich Medicine, utilize a centralized application service called the Ontario Medical School Application Service (OMSAS). General applicant information within the OMSAS application is agreed upon by all six schools, and includes name, gender (male, female, undeclared), graduate degree status. Other voluntary diversity data such as race or SES had not been traditionally captured at the application stage. Instead, once accepted into the MD program, students were surveyed for parameters such as race, SES, disability status, and LGBTQ2S+ identity by the school itself. Thus, the committee recognized that there was a large data gap in its ability to identify barriers from the application stage to the admission stage for applicants from certain URM populations.

Unlike the Indigenous Admissions pathway which holistically assessed academic metrics and non-academic experiences/attributes pre-interview, applicants through the general admissions and SWO pathways were invited for interviews based on academic metrics (GPA and MCAT scores) as published on the Schulich Medicine website (33). For several application cycles, the GPA threshold was 3.70 (equivalent to a grade of 80-84% on the OMSAS grade conversion scale) (34); the MCAT thresholds were set at the 85^th^ to 95^th^ percentile for the general admissions pathway with flexibility granted to SWO pathway applicants who must achieve at least the 50^th^ percentile for the individual MCAT sections. Recognizing variations in median MCAT scores based on diversity parameters such as race, SES, gender, and community origin, the committee identified the need to develop a more holistic assessment model for applicants through these non-Indigenous admissions pathways (35).

Schulich Medicine utilizes a panel interview comprised of 3 interviewers (a physician, community member, and senior medical student) who do not have access to other applicant information (GPA, MCAT scores, reference letters). The interview is conducted using standardized questions and scoring. While data exists that multiple mini-interviews (MMIs) may have higher inter-rater reliability compared to traditional interviews, data regarding the impact of MMIs on certain URM populations as well as internal data on good inter-rater reliability of the current interview format dissuaded the committee from switching to MMIs (36–39). Although interviewers on each panel were to score applicants independently in order to avoid groupthink bias, the committee examined methods to mitigate bias further, particularly through implicit bias awareness training. The Schulich Medicine admissions committee used this critical review to develop and implement a multipronged approach to address the gaps identified.

### Implementation of a Multipronged Approach to Enhancing EDI – Five Key Initiatives

From its critical review, the admissions committee identified the following goals of improvement for the admissions process: 1. Gathering more diversity data particularly at the applicant stage to determine whether barriers existed through the admissions process to specific URM populations; 2. Mitigating implicit biases within the admissions process; 3. Expanding the diversity of the evaluator pool and committee to create a more inclusive environment; 4. Creating a more holistic admissions process that addresses non-academic attributes, values and experiences as well as mitigates barriers for the defined URM populations.

**Figure 1:**
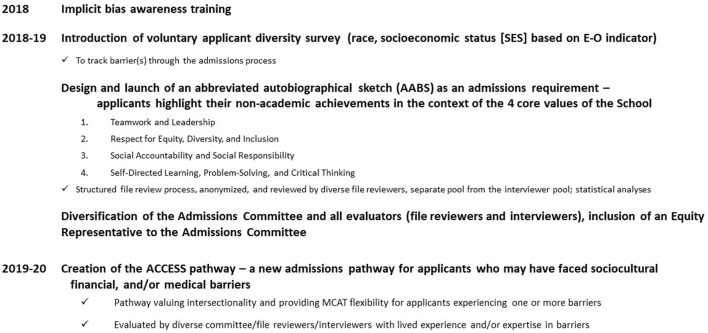
Initiatives implemented for enhancing EDI in the Schulich Medicine Admissions process.

A multipronged approach using five key initiatives was implemented from 2018 to 2020 in order to address these areas (Figure 1). First, mandatory implicit bias training was immediately instituted for committee members and evaluators in 2018, focused on self-assessment of individual and collective biases related to medical school applicants, the various forms of implicit bias, and methods to mitigate these biases (32). Second, the admissions committee (~30 members) and the evaluator pool (~600–700 people) were broadly diversified to encourage differing perspectives, mitigate biases in policy development and evaluation, and to encourage a more inclusive environment (31). Notably an Equity Representative formally trained in equity principles was specifically recruited to the admissions committee. Limits on term memberships for the committee members were also implemented to stimulate opportunities for new individuals and ideas.

Third, in the 2018–2019 application cycle, to encourage a more holistic assessment of applicants rather than strong reliance on academic metrics for interview invitations, a biosketch was instituted which was based on Schulich Medicine's core values: a. Teamwork and leadership; b. Respect for EDI; c. Social accountability and social responsibility; and d. Higher learning skills (self-directed learning, problem-solving, and critical inquiry). Applicants described a maximum of two activities/life experiences that embodied each core value, reusing activities for another section as needed. The biosketch also included an optional section for applicants to elaborate on any specific barrier(s) along their journey to medical school in order to identify distance traveled. Additional diverse faculty and community member volunteers were recruited to participate as file reviewers; the file reviewer pool was separate from the interviewer pool to minimize bias. Although concerns were raised that recruiting additional evaluators would be difficult, there was an overwhelmingly positive response for participation with community and faculty engagement more than doubling within 2 years. Several steps were taken to facilitate standardization in evaluation, including implicit bias awareness, a structured scoring system with file reviewers blinded to points allocation, distribution of anonymized files (no gender or name), and the use of a blinded test file (completed by all file reviewers) to determine statistical criteria for identifying discrepant scoring for any given applicant. Importantly, file reviewers also had no access to other aspects of the applicant file (e.g., GPA, MCAT scores, reference letters) to minimize the halo effect.

Fourth, also in the 2018–2019 application cycle, a voluntary applicant diversity survey was launched within the Schulich Medicine application to assess certain URM populations within the applicant pool to Schulich Medicine. Given limitations encountered, three priority diversity parameters were set for initial capture: a. race as defined by Statistics Canada (40); b. community origin based on population size (underserved populations of interest being those from small towns (population 10,000–49,999); rural (population of 1,000–9,999) and remote (population of < 1,000); and c. SES, using the validated AAMC parental education-parental occupation (E–O) indicator (41). To our knowledge, Schulich Medicine became the first Canadian medical school to be able to longitudinally track these diversity data from the application stage until admissions decisions were completed, to identify whether there were barriers to these URM populations through the admissions process in spite of the implemented initiatives. Applicants were reassured that these diversity data were not being used to make admission decisions nor fulfill quotas but rather to inform where barriers and gaps existed in the admissions process to make improvements for future cycles in a timely manner. Applicants demonstrated a positive response to the inclusion of the voluntary survey with response rates of at least 70–75%.

And finally, in 2019–2020, recognizing that several populations (e.g., racialized, LGBTQ2S+, PWD, and those with socioeconomic and/or family/life challenges) may still encounter barriers through the admissions process, the committee implemented the ACCESS admissions pathway, modified from a similar one within Ontario law schools. This pathway aimed at mitigating barriers for applicants who may have faced financial, sociocultural, and/or medical barriers and focused on valuing intersectionality rather than creating individual pathways separately for each possible URM population. Those who applied through the ACCESS pathway submitted a description of their barrier(s) and any supporting documentation; they completed the biosketch and interview components of the application just as other applicants to Schulich Medicine. No quotas were used. The benefits of applying through the ACCESS pathway were 2-fold: 1. ACCESS applicants were reviewed and interviewed by a panel of evaluators having lived experience and/or expertise with the barrier(s) presented; and 2. given the differences in median MCAT scores for applicants from URM populations, representing often the impact of societal inequities, MCAT flexibility could be granted to ACCESS applicants similar to the MCAT flexibility granted to SWO pathway applicants (35, 42, 43).

### Evaluation of the Multipronged Approach on Enhancing EDI

We examined the impact of these initiatives implemented from 2018 to 2020 on the diversity metrics of the medical student class that entered in the fall of 2020 (Class of 2024). In particular, we aimed to 1. compare the diversity metrics captured at the application stage to those of the admitted Class of 2024 to identify the impact of the initiatives on barriers for the URM populations captured; and 2. evaluate changes in diversity metrics from the Class of 2022 (pre-initiatives) to the Class of 2024 (post-initiatives). Data on gender (male, female, undeclared), graduate degree status, those who applied through the ACCESS pathway will have 100% response rates as these are mandatory within the OMSAS application. The remaining diversity variables were captured through survey collection administered at either the applicant stage (race, socioeconomic status, and community origin) through OMSAS or at the admitted stage through Schulich Medicine (LGBTQ2S+, language diversity, first generation Canadian, mature students, first generation to attend university, and first generation to attend medical school).

Statistical analyses were completed using SAS JMP software, version 8.0.1 (SAS Institute, Cary, NC). Pearson's Chi-squared tests were used for comparisons, and a *p* < 0.05 was considered statistically significant.

This retrospective study was approved by the Western University Ethics Review Board (Protocol #118380).

## Results

During the 2019–2020 application cycle, there were a total of 2,056 applications with 171 students admitted into the Schulich Medicine MD Class of 2024. Survey response rates for the applicants as well as for the admitted students were very similar, with 70–75% response rates for the diversity parameters of race and socioeconomic status and ~95% response rate for community origin (population of interest being those from small town, rural, and/or remote origins). For the Class of 2024, the percentage of admitted racialized students (55.2%), those with socioeconomic challenges (32.3%), and those from remote/rural/small town communities (18.6%) reflected applicant pool demographics (52.8, 29.9, and 17.2%, respectively). Additionally, 5.3% (vs. 5.6% applicant pool) of admitted students had applied through the ACCESS admissions pathway during its inception year. These data suggest that barriers were potentially mitigated and no new barriers were likely posed by the introduction of these initiatives for the URM populations captured (Table 1).

**Table 1:** Diversity parameters tracked from applicant stage to admitted stage for the Class of 2024.

	**Applicants**	**Admitted**	
	**(*N* = 2,056)**	**(*N* = 171)**	***P*-value**
Declared gender (F:M ratio)	1.4:1	1.1:1	0.09
ACCESS %	5.6	5.3	0.85
Graduate students, %	23.9	27.5	0.29
Racialized %*	52.8	55.2	0.55
Socioeconomic challenge (low SES) (%)*†	29.9	32.3	0.58
Rural, remote, small town (%)**	17.2	18.6	0.65

*F, female; M, male; SES, socioeconomic status*.

^*^Based on voluntary survey data with 70–75% survey response rate.

^**^Based on voluntary survey data with 95% survey response rate.

*^†^Based on the parental education-parental occupation (E-O) indicator (41)*.

Since applicant diversity data was not captured for the Class of 2022 (pre-initiatives), the efficacy of the initiatives implemented over the 2 years could only be examined by comparing the diversity metrics captured in the admitted classes (Class of 2024 vs. Class of 2022). Survey response rates for both classes for most diversity parameters were ~70–75% except for community origin (small town, rural, remote origins) which had ~95% response rates. Approximately 82–83% of both classes were first generation to attend medical school, and 19% within the Class of 2024 were the first in their family to attend university. Unfortunately, no comparative data existed on first generation to attend university for the Class of 2022.

The initiatives demonstrated improved representation of racialized students, LGBTQ2S+, mature students, and those with disability by ~18, 24, 28, and 88%, respectively (Table 2). Socioeconomic status was examined in the Class of 2022 using two different parameters [household income (survey response rate 70%) and the AAMC E-O indicator (survey response rate 51%)] while socioeconomic status in the Class of 2024 was examined strictly using the E-O indicator [survey response rate 72%]. Despite the lower response rate in the Class of 2022 using the E-O indicator, the initiatives implemented demonstrated a statistically significant increase in representation of students with a socioeconomically challenged background by 67% (*p* = 0.04). Similarly, language diversity (% who spoke a first language other than English and/or French) demonstrated a statistically significant increase within the Class of 2024 by 20% (*p* = 0.04). Thus, within 2 years of implementing this multipronged approach to enhancing EDI, positive increases were effected in several URM populations within the incoming medical student class at Schulich Medicine.

**Table 2:** Diversity parameters for the Class of 2024 (post-initiatives) vs. Class of 2022 (pre-initiatives).

	**Class of 2022**	**Class of 2024**	
	**(*n* = 171)**	**(*n* = 171)**	**% change**
Application data			
*Declared gender (F:M ratio)*	1:1	1.10:1	*10.0*
ACCESS %	-	5.3	-
*Graduate students, %*	20.0	27.5	*37.5*
Survey data			
Mature students %*†	19.7	25.3	*28.4*
First generation Canadian (%)*	31.1	32.6	*4.8*
Racialized %*	46.9	55.2	*17.7*
First language other than English/French*	35.0	42.1	*20.2∑*
LGBTQ2S+%*	8.9	11.0	*23.6*
Socioeconomic challenge (low SES) (%)*	10.1/19.3**/19.3∓	32.3∓	*67.3∑*
Rural, remote, small town (%) €	-	18.6	-
Disability (%)*	2.4	4.5	*87.5*
First generation in university (%)*	-	19.0	*-*
First generation in medical school (%)*	82.0	82.7	*0.8*

*F, female; M, male; LGBTQ2S+, lesbian, gay, bisexual, trans, queer or questioning, two-spirited; SES, socioeconomic status*.

*^†^Mature students are defined as those aged 25 years or older*.

^*^Based on voluntary survey data with 70–75% survey response rate.

*>^€^ Based on voluntary survey data with approximate 95% response rate*.

^**^For the class of 2022, 2 separate surveys with differing definitions of socioeconomic status had been conducted and both are presented for readers. The survey with a 70% response rate defined low SES based on household income where 10.1% of the incoming student class had a household income < $60,000 and 19.3% had a household income of < $80,000.

*^∓^The second survey for the Class of 2022 (51% response rate) and the survey used for the Class of 2024 (72.5% response rate) defined SES using the parental E-O indicator (41)*.

*∑ p = 0.04*.

## Discussion

Schulich Medicine utilized a multi-pronged approach to enhancing EDI broadly within its medical school admissions process. With the five initiatives implemented in Schulich Medicine's non-Indigenous MD admissions pathways (Figure 1), positive increases in a variety of diversity parameters (female gender, mature students, racialized, LGBTQ2S+, those with disabilities) and statistically significant increases in socioeconomic diversity and language diversity were evident. Importantly, the admitted Class of 2024 reflected the available diversity metrics of the applicant pool, indicating that barriers to the URM populations examined were potentially mitigated and no new barriers were likely introduced with this approach. Thus, the multipronged approach was helpful in diversifying the incoming medical student cohort within 2 years of implementation, and given the low attrition rates amongst Canadian medical schools, this diversification will translate into improvements in the diversity of the future Canadian physician workforce (44, 45).

The multipronged approach addressed two broad areas–mitigation of implicit bias and institution of a more holistic assessment. Since admissions is a high-stakes process, implicit bias awareness training as well as diversification of the admissions committee members and evaluators have been highlighted as important strategies for improving diversity within the medical school cohort (31, 32). Schulich Medicine also mitigated biases throughout the admissions process by ensuring file reviewers and interviewers had no access to other aspects of the applicant's files (GPA, MCAT, biosketch, reference letters), using de-identified biosketches for file reviewers, increasing the number of evaluators for a given applicant with separate file reviewer and interviewer pools, and utilizing a structured scoring system and blinded test file to determine acceptable statistical variation for file reviews. With the implementation of a biosketch and the ACCESS admissions pathway, Schulich Medicine assessed applicants using a more holistic process, valuing their experiences, attributes, and metrics including distance traveled (29, 30). However, this required care in training evaluators particularly for the ACCESS admissions pathway, and the specific inclusion of individuals with lived experiences and/or expertise in the barrier(s) presented by applicants helped in not only mitigating biases but also potentially removing barriers to medical school for these applicants.

The increasing numbers of female matriculants over the 2 years was expected given the trend in applicants and matriculants within Canada and may not be completely due to the changes implemented (46). Unfortunately, gender captured on the OMSAS application was strictly limited to male, female and undeclared. Increased representation of those who are LGBTQ2S+ and those with disabilities within the Class of 2024 is promising, particularly considering that stigma may still be a barrier to disclosure and physician role models for these and other URM populations are still lacking (47). Some medical students have disclosed disability for the purposes of countering stigma and ableist conceptions within medical education, thereby aiming to shift the view of the medical profession away from the traditional biomedical view of disability as a pathology, impairment or dysfunction to a social model focused on mitigating barriers and improving capabilities of PWD (48, 49). Physician bias and lack of physician knowledge regarding the needs of LGBTQ2S+ and PWD populations contribute to suboptimal health care (2, 3, 50–54). Consequently, there have been growing calls for conscientious efforts to increase the recruitment of sexual URM and PWD populations into the physician workforce, since education about LGBTQ2S+ and PWD healthcare needs through near-peer experiences in a medical school class potentially may improve medical students' comfort and attitudes about caring for these populations (48, 50).

The increases in the proportion of incoming medical students who are LGBTQ2S+ and with disabilities by 24 and 87%, respectively, within the Class of 2024 were likely partly due to the effects of these initiatives, including the specific implementation of the ACCESS pathway. However, for PWD, specific aspects in the admissions process may still influence even the decision to apply such as technical standards requirements for future medical students (55), academic requirements (e.g., full-time studies), receipt of inadequate accommodations during schooling/testing, and limitations in extracurricular participation. Capturing diversity data related to the proportion of PWD as well as those who are LGBTQ2S+ at the applicant stage would thus be helpful in understanding barriers through the admissions process as well as pre-application barriers.

The Canadian population is highly multicultural with 22% racialized, 24 % first generation Canadians and 21% speaking a first language other than the official languages of English and French (19). Patients in race-concordant patient-physician relationships rate greater satisfaction with their visits compared to those in race-discordant relationships (56). Patients with limitations in English proficiency within English-speaking countries experience higher rates of adverse medication reactions and more serious adverse events, when compared to English proficient patients and these issues are often ascribed to the patient being a “poor historian” rather than communication barriers on the part of physicians (57–59). Interestingly, patient-physician language concordance may be associated with improvement in certain health parameters such as glycemic control and mental health (60). The multipronged approach at Schulich Medicine resulted in an 18% increase in racialized students and 20% increase in language diversity within 2 years. Thus, while race and ethnicity are important considerations within the physician workforce, linguistic diversity amongst future physicians may also be critical to fostering improved cross-cultural communication, participatory decision making, and health care outcomes.

Several studies have identified overrepresentation of Canadian medical students from higher income households ($100,000 or more) when using census data as comparators (24–26). Despite limited generalizability due to the relatively low sample sizes and low inclusion of French-language medical schools within Canada, the findings are likely true but not necessarily surprising. In the United States, more robust definitions of SES beyond household income have been utilized to help identify and recruit students from socioeconomically challenged backgrounds, particularly given evidence of their willingness to serve populations with similar backgrounds (16, 41, 61). Interestingly, 83% of the Class of 2024 were the first in their family to attend medical school, which was not drastically different from the Class of 2022 (82%), challenging the myth that students of physician parent(s) comprise the vast majority of students in a medical school class and yet, the proportion of medical students with socioeconomic challenges, based on the AAMC E-O indicator, increased significantly by 67% within 2 years, representing 32% of the Schulich Medicine Class of 2024. The initiatives therefore had the most significant effect on socioeconomic diversity of the medical school class.

The goal of creating a diverse physician workforce is to better serve the needs of the population, and while the workforce should be representative, it does not necessarily mean that the demographic breakdown of the medical student cohort should reflect the demographic makeup of a country or region exactly, as per census data. Doing so would be a difficult feat for medical schools to achieve fairly. Increasing the recruitment of individuals from URM populations can not be merely “fixed” at the medical school admissions level since this approach does not address the larger societal inequities, especially those occurring even before students consider applying to medical school. Within Canada, admissions requirements for medical school often value undergraduate years at a university (not college), require pre-requisites with or without the MCAT, expect full-time studies, and may even require completion of a degree. In addition, since service to others is considered a desired characteristic, commitment to volunteering, charities, or other non-profit activities with or without physician shadowing can be highly emphasized and may even be highly valued within admissions processes, resulting in perpetuation of inequities. Although there have been calls to remove standardized testing in higher education for equity purposes, the representation of URM medical students within schools in Canada that currently do not use the MCAT vs. those that do is actually not known. Requirements for yearly situation judgment tests only add further to the burden for applicants.

Living costs and tuition associated with medical school are large barriers that influence the decision just to apply to medical school even when application/MCAT testing fee waivers are provided or free application/interview support is provided (62, 63). Geographic barriers to participating in activities valued by admissions committees may disproportionately affect those from small towns and rural communities. Similarly, there may be expectations to enter the workforce earlier which may be a consideration for those with family and/or socioeconomic challenges, those of certain ethnicities, and those who do not have parental or other familial role models with higher education attainment. Thus, while the initiatives implemented within Schulich Medicine's non-Indigenous admissions pathways demonstrated positive gains in a number of URM populations, a more comprehensive approach to mitigating barriers earlier in the journey to medical school (pre-application) through to once accepted (post-admission) are required by all universities and the government in order to truly diversify the physician workforce.

This study has a few limitations. First, this study is at a single medical school within Canada and thus, results may not be generalizable to other regions since diversity priorities and feasible solutions will need to be aligned with local context and any relevant laws. Similarly, this study was designed to address potential barriers within the admissions process itself, and therefore cannot address any pre-application barriers as outlined above. However, this study did aim to provide practical initiatives (Figure 1) that could be applied and modified for most medical school admissions departments, including those where affirmative action efforts such as race-conscious admissions may not be possible. Second, the main outcomes of the study focused on the impact of the multipronged approach within a relatively short period of time. While the approach did demonstrate positive changes in several diversity parameters within 2 years, achieving statistically significant improvements in all diversity parameters in this short timeframe would have been impossible. The statistically significant improvements in socioeconomic diversity and language diversity are promising for other diversity parameters also to likely reach significance with a longer timeframe. Thus, the longer-term impact, including any benefits and/or unintended effects (such as creation of new barriers), remain unknown. Furthermore, whether these changes in diversity within the medical student cohort translate into health outcome benefits for underserved patient populations will need to be examined. Third, the definition of URM population used within this study was very broad and differs from other studies that define URM on race. The study specifically utilized a multipronged approach with initiatives from the literature (29–32) as well as initiatives developed for local context designed to value intersectionality and advance multiple URM populations. Thus, it is not possible to identify which of the initiatives had the greatest and/or least impact on diversifying the incoming medical school class. The study also did not examine the Indigenous admissions pathway given its already holistic approach, but efforts to evaluate that pathway would likely be beneficial. And finally, the study did not have robust applicant data related to gender identity, sexual identity, disability status, first generation Canadian, first generation in university. This data will be helpful to dissecting the admissions process more critically for these URM populations for this approach to be constantly evaluated and modified for efficacy and fairness as well as evaluating the impact on the diversity of the applicant pool.

This study revealed that the initiatives implemented increased diversity within the incoming medical school Class of 2024 across a variety of URM populations within 2 years of Schulich Medicine's multipronged approach to enhancing EDI. Valuing intersectionality and harnessing the power of a holistic assessment and methods to mitigate bias at multiple points through the admissions process helped to garner these positive effects. Future efforts can focus on engaging in a continuous quality improvement method to enhancing EDI further within admissions. This will involve gathering more applicant diversity data for parameters that were not captured (e.g., sexual identity, disability status), examining specific sub-populations (e.g., Black, Filipino, Indigenous), assessing longer-term effects of the approach implemented, and understanding where barriers may be occurring (pre-application vs. admissions) to determine modifications to existing initiatives and/or development of new initiatives. Since each medical school has varying selection practices, tracking the impact of each of their selection practices on the journey of the applicant pool for locally-determined URM populations of interest should be made an expectation within the social accountability mandate of each medical school in order to diversify the future physician workforce and improve health equity effectively.

## Data Availability Statement

The datasets presented in this article are not readily available because the data pertains to medical student diversity parameters, and is meant to remain confidential. Requests to access the datasets should be directed to tisha.joy@sjhc.london.on.ca.

## Ethics Statement

This study received approval by Western University REB #118380. Written informed consent for participation was not required for this study in accordance with the national legislation and the institutional requirements.

## Author Contributions

The author confirms being the sole contributor of this work and has approved it for publication.

## Conflict of Interest

The author declares that the research was conducted in the absence of any commercial or financial relationships that could be construed as a potential conflict of interest.

## Publisher's Note

All claims expressed in this article are solely those of the authors and do not necessarily represent those of their affiliated organizations, or those of the publisher, the editors and the reviewers. Any product that may be evaluated in this article, or claim that may be made by its manufacturer, is not guaranteed or endorsed by the publisher.
